# Simulating the Impact of Future Climate Change and Ecological Restoration on Trade-Offs and Synergies of Ecosystem Services in Two Ecological Shelters and Three Belts in China

**DOI:** 10.3390/ijerph17217849

**Published:** 2020-10-26

**Authors:** Liang-Jie Wang, Shuai Ma, Yong-Peng Qiao, Jin-Chi Zhang

**Affiliations:** 1 Co-Innovation Center of Sustainable Forestry in Southern China, Jiangsu Provincial Key Lab of Soil Erosion and Ecological Restoration, Nanjing Forestry University, Nanjing 210037, China; ms19961103@outlook.com (S.M.); zhang8811@njfu.edu.cn (J.-C.Z.); 2 School of Computer Science and Engineering, Northeastern University, Shenyang 110006, China; ali_qiao@163.com

**Keywords:** ecosystem services, climate change, ecological restoration, trade-offs/synergies, sustainable management

## Abstract

Development of suitable ecological protection and restoration policies for sustainable management needs to assess the potential impacts of future land use and climate change on ecosystem services. The two ecological shelters and three belts (TSTB) are significant for improving ecosystem services and ensuring China’s and global ecological security. In this study, we simulated land use in 2050 and estimated the spatial distribution pattern of net primary productivity (NPP), water yield, and soil conservation from 2010 to 2050 under future climate change. The results showed that water yield, NPP, and soil conservation exhibited a spatial pattern of decreasing from southeast to northwest, while in terms of the temporal pattern, water yield and NPP increased, but soil conservation decreased. Water yield was mainly influenced by precipitation, NPP was affected by temperature and implementation of ecological restoration, and soil conservation was controlled by precipitation and slope. There was a strong spatial heterogeneity between trade-offs and synergies. In terms of the temporal, with the combination of climate change and ecological restoration, there was a synergistic relationship between water yield and NPP. However, the relationships between water yield and soil conservation, and between NPP and soil conservation were characterized by trade-offs. In the process of ecological construction, it is necessary to consider the differences between overall and local trade-offs and synergies, as well as formulate sustainable ecological management policies according to local conditions. Understanding the response of ecosystem services to future climate change and land use policies can help address the challenges posed by climate change and achieve sustainable management of natural resources.

## 1. Introduction

Ecosystem services are the benefits that ecosystems provide to sustain human existence and development, thereby linking the natural environment and human well-being together [1,2]. Recently, the study of ecosystem service trade-offs and synergies has attracted the attention of policy makers [3,4,5]. Trade-offs are defined as the increase in one or more ecosystem services at the expense of other ecosystem services, while synergies refer to the simultaneous increase or decrease in two or more ecosystem services [6,7]. Quantifying ecosystem services and revealing their relationships are beneficial to policy decisions and the sustainable management of ecosystem services [8,9,10,11]. Climate change and land use are considered to be two main factors affecting ecosystem services and their trade-offs and synergies [12,13,14]. In recent years, ecosystem services have been seriously threatened by climate change and socioeconomic development [15,16,17]. Changing trends in land use pattern will pose a serious threat to future ecological security. In addition, climate change may have a significant impact on ecosystems, thereby leading to the disruption of ecosystem services and seriously threatening the living environment of human beings and the sustainable development of social economy [18,19,20]. Many studies only considered a single factor and ignored the common impacts of multiple driving factors on ecosystem services, such as global climate change and land use change [21,22,23]. Therefore, understanding the impacts of the future climate and land use on ecosystem services and their trade-offs and synergies is the scientific basis for climate change mitigation and adaptation.

Ecological restoration projects often improve ecosystem services by changing land use [11]. Since 2000, China has conducted large-scale ecological restoration projects, such as the Nature Forest Protection Program and Grain for Green Program (GFGP) [24,25,26,27]. Ecological restoration aims to restore damaged ecosystems by turning farmland into grassland and protecting vegetation cover [28]. A large number of studies have shown that ecological restoration has a significant impact on ecosystem services [29,30,31,32]. Wang showed that the implementation of the GFGP increased soil conservation but reduced net primary productivity (NPP) and water yield [33]. Jia indicated that there was a trade-off between the supply service and regulation service [34]. Some scholars have assessed future ecosystem services under ecological restoration. Peng explored the impact of different GFGP intensities on the trade-offs of future ecosystem services without taking into account future climate change [11]. Wen and Theau assessed future water yield and soil conservation under ecological restoration [28]. However, they only studied future ecosystem services under the Representative Concentration Pathway (RCP) 4.5 scenario. So far, most studies have focused on short-term effects, but ecological processes tend to occur over relatively long periods of time [35,36,37]. Thus, clarifying trade-offs and synergies between ecosystem services on a medium or long-term scale under different RCP scenarios can help enhance and optimize regional ecosystem services and can provide an important basis for sustainable development [38]. Therefore, it is necessary to study the impacts of future climate change and ecological restoration on ecosystem service trade-offs and synergies under different RCP scenarios. The RCP was proposed by the Coupled Model Intercomparison Project Phase 5 (CMIP5) and could describe various future climate scenarios [39,40]. RCP scenarios describe the trajectories of greenhouse gas and pollutant concentration changing with time and space. RCP scenarios include RCP2.6 (a very low forcing scenario), RCP4.5 and 6.0 (a medium stability scenario), and RCP8.5 (a very high emission scenario). The greenhouse gas emission level in the RCP4.5 scenario is more in line with the trend of China’s future economic development, while the RCP8.5 represents a high-risk future scenario [41]. Both scenarios are widely used in studies of the impact of future climate change on ecosystem services [15,28,42].

China has one of the most fragile ecological environments worldwide, and the irrational use of resources has led to a series of ecological problems such as forest and grass degradation, land desertification, and serious soil erosion [25,43,44]. In order to improve the environment and maintain the national ecological security, the “two ecological shelters and three belts” (TSTB) national ecological security shelter framework was put forward. The TSTB are one of the most important ecological system protection and restoration regions in China. The protection and restoration of TSTB are regarded as important tasks to accelerate ecological civilization construction, national ecological security, and realize harmonious coexistence between man and nature. In recent years, the change of ecosystem pattern has been seriously disturbed by human beings and ecological zoning. Environmental governance still has great blindness and uncertainty, and ecological security guarantee has become an urgent social demand. The changes of ecosystem pattern of the TSTB from 2000 to 2015 reflected the influence of ecological restoration on ecosystems [45]. Fu determined the boundaries of national shelter areas and conducted a comprehensive assessment of ecosystem services from 2000 to 2010 [44]. The research showed that ecosystem services improved with the implementation of ecological restoration. Yin explored the trade-offs and synergies between soil conservation, NPP and water yield in National Barrier Zone from 2000 to 2015 [46]. In order to further consolidate and enhance regional ecological service and ensure national ecological security, the 2018 National Key Project “Research on Restoration and Protection of Typical Fragile Ecosystems” clearly pointed out that the ecosystem service pattern of TSTB should be optimized, and the optimization of ecosystem services cannot be achieved without an accurate understanding of trade-offs and synergies [46]. In the context of global climate change, the future ecosystem services in the TSTB are subject to great spatial and temporal uncertainty. The study of ecosystem service trade-offs and synergies under future climate change is beneficial for policy makers to develop sustainable ecosystem management strategies under global changes and is of great significance to the maintenance of national ecological security.

In this study, the TSTB were selected as the study area. We simulated land use in 2050 using the Future Land Use Simulation (FLUS) model, and the Carnegie–Ames–Stanford Approach (CASA) model and Integrated Valuation of Ecosystem Services and Trade-offs (InVEST) model were applied to evaluate the spatial distribution pattern of NPP, water yield and soil conservation from 2010 to 2050 under the RCP4.5 scenario and the RCP8.5 scenario. Finally, the changes in ecosystem services and the trade-offs and synergies of different ecosystem services were quantified. The specific objectives were to (1) quantify the water yield, soil conservation and NPP using the InVEST model and CASA model; (2) assess the impact of future land use changes and climate change on ecosystem services; (3) quantify the trade-offs/synergies among ecosystem services and analyze their spatiotemporal distribution characteristics; (4) propose different sustainable management strategies of each shelter area according to local condition. The results can provide a scientific reference for decision makers to make better ecological protection policies.

## 2. Materials and Methods

### 2.1. Study Area

**Figure 1:**
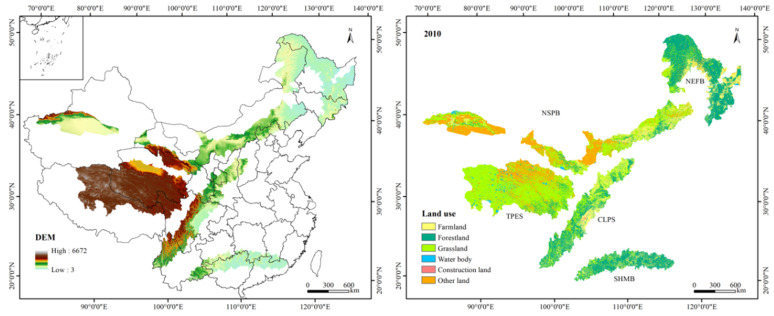
Geographical overview and spatial distribution of digital elevation model (DEM) and land use types of the two ecological shelters and three belts (TSTB).

The TSTB are composed of the southern hill and mountain belt (SHMB), the Chuan-Dian and Loess Plateau ecological shelter (CLPS), the Tibet Plateau ecological shelter (TPES), the northeast forest belt (NEFB) and the northern sand prevention belt (NSPB) (Figure 1). The TSTB are made up of nearly 500 counties, with a total area of 3.16 million km^2^. Farmland, grassland, and forestland are the main land use types. The elevation ranges from 3 m to 6672 m and decreases from southwest to northeast. Precipitation is high in the southeast and low in the northwest, with an average temperature between −6 °C and 23 °C. The terrain of the SHMB is complicated, the precipitation is abundant, and the soil erosion problem is serious. The CLPS is an important GFGP region with serious soil erosion. Grassland is the main land use type in the TPES, and forestland is the main land use type in the NEFB. The NSPB is a typical agropastoral ecotone with low vegetation coverage.

### 2.2. Data Collection

The 1 km × 1 km digital elevation model (DEM) was acquired from the National Tibetan Plateau Data Center (http://data.tpdc.ac.cn). Land use data in 2000 and 2010 with a spatial resolution of 1 km were obtained from the Data Center for Resources and Environmental Sciences, Chinese Academy of Sciences (http://www.resdc.cn/). The normalized vegetation index (NDVI) data, gross domestic product (GDP) and population spatial distribution data (POP) were also obtained from the Data Center for Resources and Environmental Sciences, Chinese Academy of Sciences (http://www.resdc.cn/). Furthermore, the future NDVI data were obtained from future vegetation datasets (http://data.ess.tsinghua.edu.cn). Meteorological data were downloaded from the Meteorological Administration of China (http://data.cma.cn/) and Beijing Normal University Earth system model (BNU-ESM) data were obtained from the Coupled Model Intercomparison Project Phase 5 (CMIP5) climate prediction downscaling dataset (http://stdown.agrivy.com/) [47]. In order to avoid the impact of extreme climate and reflect the results more accurately, the meteorological data used in this study were the average climatic data of 20 years. Kriging interpolation method was used to obtain the precipitation and temperature maps. A soil type map was obtained from the Cold and Arid Regions Sciences Data Center (http://www.westdc.westgis.ac.cn/). City, railway, road, and river data were from the National Earth System Science Data Center (http://www.geodata.cn/).

### 2.3. Quantification of Ecosystem Services

**Figure 2:**
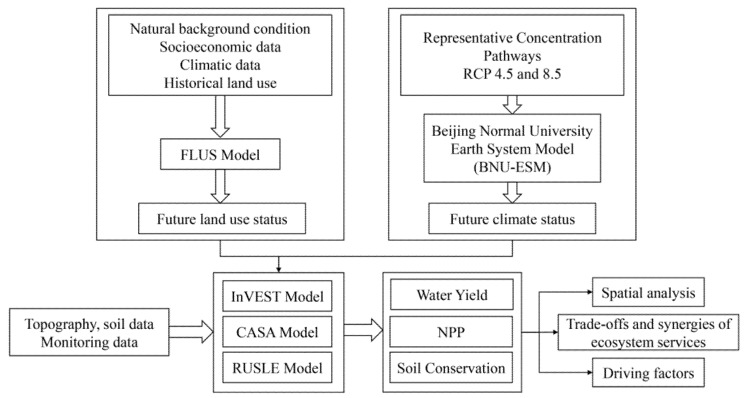
Framework for assessing trade-off and synergies among multiple ecosystem service responses to future climate change and land use change.

We proposed a framework for coupling land use model, climate model and ecosystem services model to assess trade-off and synergies among multiple ecosystem service responses to future climate change and ecological restoration (Figure 2).

#### 2.3.1. Water Yield

The Integrated Valuation of Ecosystem Services and Trade-offs (InVEST) model considers the influence of topography on runoff and the spatial differences of soil permeability under different land use types, and quantitatively estimates the water yield of different land use types based on the principle of water balance [48], as follows:

(1) $$Y_{xj}=(1-\frac{AET_{xj}}{P_{x}})\cdot P_{x}$$

where $Y_{xj}$ refers to the average water yield of pixel *x* for the land use type *j*, $AET_{xj}$ is the actual evapotranspiration on the pixel *x* with land use type *j*, and $P_{x}$ represents the annual precipitation.

(2) $$\frac{AET_{xj}}{P_{x}}=\frac{1+\omega_{x}R_{xj}}{1+\omega_{x}R_{xj}+\frac{1}{R_{xj}}}$$

where $R_{xj}$ refers to the Budyko index of dryness of pixel *x* for land use type *j* and $\omega_{x}$ is the ratio of the available water content of vegetation to precipitation.

(3) $$R_{xj}=\frac{k_{xj}ET_{0x}}{P_{x}}$$

where $k_{xj}$ refers to the evapotranspiration coefficient of pixel *x* for land use type *j* and $ET_{0x}$ is the potential evapotranspiration (mm) of pixel *x*.

(4) $$\omega_{x}=Z\cdot \frac{AWC_{x}}{P_{x}}$$

where *Z* refers to the seasonal factor and $AWC_{x}$ is the available water content of vegetation.

(5) $$AWC_{x}=\min(MaxSoilDepth_{x},RootDepth_{x})\cdot PAWC_{x}$$

where $MaxSoilDepth_{x}$ refers to the maximum depth of the soil for pixel *x*, $RootDepth_{x}$ is the depth of the root system of the crops for pixel *x* and $PAWC_{x}$ is the effective water content of the vegetation for pixel *x*.

The input parameters and calculation methods of the water yield model referred to the study of Sun and Yang [49,50].

#### 2.3.2. Soil Conservation

The sediment transport ratio module in the InVEST model calculates soil conservation based on the revised universal soil loss equation (RUSLE) equation, as follows:

(6) A=R×K×L×S×(1−C×P)

where *A* is soil conservation, *R* represents the precipitation erosivity, *K* refers to the soil erodibility, *L* is the slope length factor, *S* is the slope gradient factor, *C* is the vegetation management factor, and *P* is the practice factor.

The calculation method of *R* referred to Food and Agriculture Organization of the United Nations (FAO) [51,52], the calculation method of *K* used the Erosion Productivity Impact Calculator (EPIC) formula [44,53]. *L* and *S* were calculated by the InVEST model. *C* and *P* referred to the research of Sun and Fu [44,49].

#### 2.3.3. Net Primary Productivity

NPP is an important index to evaluate regional vegetation productivity. In this study, the Carnegie–Ames–Stanford Approach (CASA) model, based on the light energy utilization principle, was used to estimate NPP [54,55], as follows:

(7) NPP(x,t)=APAR(x,t)×ε(x,t)

where APAR(x,t) is the effective photosynthetic radiation absorbed by pixel *x* in month *t*, and ε(x,t) is the actual utilization rate of light energy.

(8) APAR(x,t)=SOL(x,t)×FRAR(x,t)×0.5

where SOL(x,t) is the total solar radiation of pixel *x* of t month, FRAR(x,t) is the absorption ratio of vegetation to incident photosynthetically active radiation:

(9) $$\varepsilon (x,t)=T_{\varepsilon 1}(x,t)\times T_{\varepsilon 2}(x,t)\times W_{\varepsilon }(x,t)\times \varepsilon_{\max}$$

where $T_{\varepsilon 1}(x,t)$ is the inhibitory effect of low temperature on light utilization, $T_{\varepsilon 2}(x,t)$ is the inhibitory effect of high temperature on light utilization, and $W_{\varepsilon }(x,t)$ is the stress coefficient affected by water. The parameter input of the CASA model was referred to by Zhu [55].

### 2.4. Land Use Change and Climate Change Scenarios

The FLUS model is an improved spatiotemporal simulation model of land use based on cellular automata. The FLUS model selects natural and human driving factors by using an artificial neural network (ANN). In addition, it can well handle the transformation between various land use types and has high simulation accuracy compared with other land use models such as CLUE-S, Logistic-CA and ANN-CA [56]. Moreover, it can realize large-scale simulation. Therefore, it is widely used in regional, national and even global land use simulations [57,58,59].

We selected the DEM, slope, aspect, GDP, POP, precipitation, temperature, distance from the city, distance from the railway, distance from the highway and distance from the river as the driving factors, and calculated the probability of land use suitability based on an ANN. Firstly, we simulated the land use in 2015 and compared the actual land use to verify the model. Then, based on the predicated results of the Markov chain, we simulated the land use in 2050 based on the land use in 2010.

The RCP was presented in the fifth assessment report of the Intergovernmental Panel on Climate Change, and was often used to assess climate change [60]. In this study, we used the RCP4.5 and 8.5 scenarios of the BNU-ESM model. The Kriging interpolation method was used for all the meteorological data.

### 2.5. Statistical Analysis

Studies have shown that the Pearson correlation analysis could well identify trade-offs and synergies between ecosystem services [61,62]. Therefore, this study used the Pearson correlation analysis to assess the spatial and temporal trends of trade-offs and synergies from the perspective of the whole area and each shelter. We used ArcGIS software to randomly select 10,000 points in each shelter, and then extracted the service value of each sample point for statistical analysis in SPSS. 

## 3. Results

### 3.1. Future Land Use Change and Climate Change

**Figure 3:**
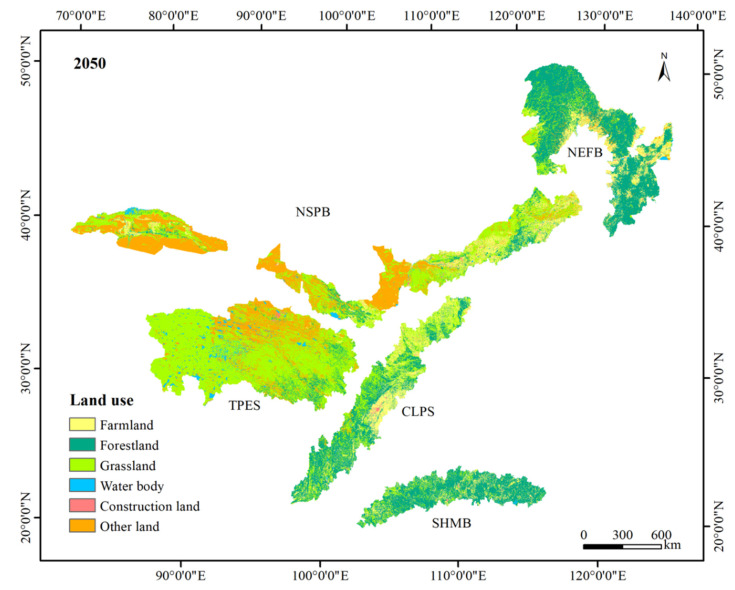
Spatial distribution of land use types in the TSTB in 2050.

The accuracy of the simulated land use map in 2015 was more than 95%, indicating a high consistency between the simulated land use and the actual land use. Figure 1 and Figure 3 show the spatial and temporal distribution of land use types. Grassland, forestland, other land, and farmland were the dominant land use types, accounting for approximately 39%, 28%, 17%, and 13%, respectively. Grassland and other land were mainly distributed in the TPES and NSPB, while forestland and farmland were mainly concentrated in the NEFB, CLPS, and SHMB. According to land use change, the grassland area decreased the most, from 1233.19 thousand km^2^ to 1217.82 thousand km^2^. The other land declined slightly by 0.02%. Grassland increased the most, followed by water bodies, which increased by 6862 km^2^ and 5263 km^2^, respectively. Farmland and construction land expanded by 0.07% and 0.06%, respectively. Conclusively, the degree of land use change in the TSTB was small and the land use pattern was stable, but we could still observe the existence of ecological restoration.

Table 1 shows the land use compositions of each shelter in 2010 and 2050. All the shelter areas were stable, but their land use structures and change patterns were different. The grassland of the TPES accounted for approximately 70%, showing a decreasing trend with a decrease of 3071 km^2^. In addition, the bare land distributed in the northern region accounted for nearly 21% of the total land. Forestland of the TPES was mainly located in the southeast. The proportions of grassland in the NEFB and SHMB were more than 60% and showed an increasing trend. The water bodies in the SHMB and the farmland in the NEFB decreased. The farmland in the CLPS accounted for approximately 26% of the total land, and the forestland and grassland accounted for nearly 71%. The trend of change was consistent with the whole area except for a slight decrease in construction land. The farmland and grassland in the CLPS were mostly located in the north and central regions. The land use change in the NSPB showed a significant difference, with a decrease in forestland and an increase in other land. The proportions of grassland and other land in the NSPB were both more than 37%, with the area of grassland decreasing by 4629 km^2^ and the area of other land increasing by 641 km^2^. The other land in the NSPB was mainly deserts, which was distributed in the Central and western regions.

**Figure 4:**
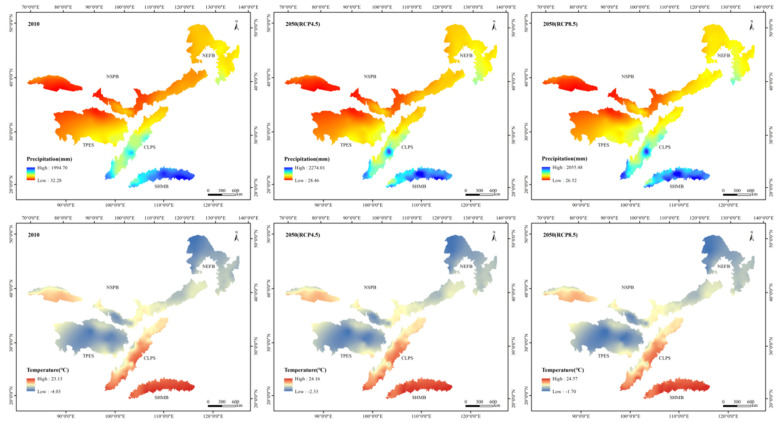
Spatial distribution of precipitation and temperature in 2010 and under Representative Concentration Pathway (RCP)4.5 and RCP8.5 scenarios in 2050.

The climate of the TSTB showed a warming and wetting trend under the RCP4.5 and RCP8.5 scenarios, especially the RCP8.5 scenario (Figure 4). The precipitation patterns between 2010 and 2050 were similar, with a decreasing trend from southeast to northwest. The high-value areas were mainly concentrated in the SHMB, while the precipitation in the NSPB was low. The average precipitation in the TSTB in 2010 was 559.60 mm, and the values under the RCP4.5 and RCP8.5 scenarios in 2050 increased by 3.7% and 4.3%, respectively. However, the variation in precipitation in each ecological shelter showed different trends. Compared with 2010, the average precipitation in the TPES showed a decreasing trend under the RCP4.5 scenario, while it showed an increasing trend under the RCP8.5 scenario. The average precipitation in the NSPB increased under both RCP scenarios, especially under the RCP8.5 scenario. The average precipitation in the NEFB also showed an increasing trend under both RCP scenarios, but the increase in the RCP4.5 scenario was larger. The precipitation in the SHMB under the RCP4.5 scenario slightly reduced by 1.08 mm, while it reduced by 104.21 mm under the RCP8.5 scenario. The variation in precipitation in the CLPS showed a consistent trend with that in the TPES. The increase in average precipitation in the CLPS in the RCP8.5 scenario was 9.63 mm, while in RCP4.5 scenario, the average precipitation decreased by 3.86 mm. The temperature in the TSTB was high in the south and low in the north. Low temperatures were found in the TPES and NEFB, while the temperature is higher in the SHMB. The average temperature in the TSTB in 2010 was 7.01 °C, while that under the RCP4.5 and RCP8.5 scenarios in 2050 increased by 1.42 °C and 2.21 °C, respectively. The average temperature in each shelter showed an upward trend under both RCP scenarios, and the temperature increased more under the RCP8.5 scenario. Among them, the average temperature of the TBES increased the most, and those in the RCP4.5 and 8.5 scenarios increased by 1.56 °C and 2.59 °C, respectively. Moreover, the average temperature in the SHMB minimally increased, rising from 20.15 °C to 21.16 °C and 21.63 °C under the RCP4.5 and RCP8.5 scenarios, respectively.

### 3.2. Temporal and Spatial Changes in Ecosystem Services

**Figure 5:**
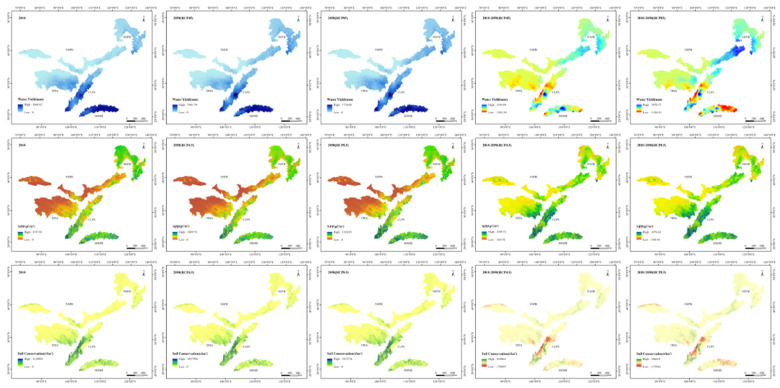
Spatial distribution and the difference between 2010 and 2050 of water yield, NPP and soil conservation in the TSTB.

We compared the simulated ecosystem services in 2010 with the results of Yin [46], and the overall results were similar, indicating that the simulated results were credible. The spatial and temporal distribution of water yield, NPP and soil conservation in the TSTB are shown in the Figure 5. All the ecosystem services showed a pattern of high in the southeast and low in the northwest. The high-value areas were concentrated in the CLPS and SHMB, with the intermediate values in the NEFB and the southeastern part of the TPES, and the low-value areas located in the NSPB and the northwest of the TPES. In 2010, the average water yield, NPP, and soil conservation of the TSTB were 208.04 mm, 263.17 cg/m^2^, and 3089.00 t/km^2^, respectively (Table 2). The average water yield, NPP and soil conservation of the CLPS in 2010 were 406.47 mm, 410.87 cg/m^2^, and 11119.87 t/km^2^. Under both RCP scenarios, the water yield and NPP in the TSTB showed an increasing trend, while the soil conservation showed a decreasing trend from 2010 to 2050. Under the RCP4.5 scenario, the water yield of the TSTB increased by 27.89 mm, while it increased by 24.88 mm under the RCP8.5 scenario. The NPP of the TSTB increased by 15.17% and 15.19% in the RCP4.5 and RCP8.5 scenarios, respectively. The soil conservation of the TSTB decreased to 3078.12 t/km^2^ in the RCP4.5 scenario and 3070.17 t/km^2^ in the RCP8.5 scenario.

Under the RCP4.5 scenario, the water yield in 54.39% of the TSTB increased, and the areas with maximum increases were concentrated in the central part of the SHMB and the southwest part of the CLPS. The areas with a clear decrease in water yield were located in the middle of the CLPS. The NPP in the 72.26% of the region increased, with the largest increase distributed in the central and southwest regions of the CLPS, the SHMB, and the southeast part of the TPES. NPP declined in the northeast part of the NEFB and the western part of the NSPB. Soil conservation increased in 53.45% of the region of the TSTB, and the regions with the greatest increase and decrease in soil conservation were mainly concentrated in the central and southern parts of the CLPS.

Under the RCP8.5 scenario, the area with reduced water yield accounted for 39.24%. The areas with a decline were mainly in the eastern part of the SHMB. The areas with an increased water yield were mainly distributed in the eastern part of the NSPB, southern part of the CLPS, and western part of the SHMB. The area with an NPP decrease (28.40%) was far smaller than that with an increase (71.60%). The decreased areas were mainly scattered in the north of the NEFB, while the areas with increased NPP were mainly located in the SHMB, CLPS, and the southeast part of the TPES. The area with increased soil conservation accounted for 65.49%. The decreased areas were mainly distributed in the middle of the CLPS, while the increased areas were mainly located in the southern part of the CLPS.

The variation trends of water yield, NPP, and soil conservation were not consistent in each shelter under both RCP scenarios. Under the RCP4.5 scenario, water yield increased slightly in the CLPS, and it increased significantly in the SHMB, NEFB, and NSPB, while it decreased in the TPES. Under the RCP8.5 scenario, except for the SHMB, the water yield showed an increasing trend. The NPP in each ecological shelter increased under both RCP scenarios. Under the RCP4.5 scenario, NPP in the CLPS, SHMB and the TPES increased more, while NPP in the NEFB and the NSPB increased more under the RCP8.5 scenario. Soil conservation in the TPES decreased under the RCP4.5 scenario but increased under the RCP8.5 scenario. Soil conservation in the NEFB and NSPB increased under both RCP scenarios, among which soil conservation in the NEFB more under the RCP4.5 scenario, while soil conservation in the NSPB strengthened under the RCP8.5 scenario.

### 3.3. Trade-Offs and Synergies of Ecosystem Services

Table 3 shows the correlation coefficients among water yield, NPP, and soil conservation in 2010 and under the two RCP scenarios in 2050. The correlation coefficients of the three ecosystem services in the TSTB were all greater than 0 in 2010 and 2050, which indicated that significant synergistic relationships existed among water yield, NPP, and soil conservation (*p* < 0.01). In 2010 and 2050, ecosystem services of the TPES and NSPB showed significant synergies. NPP had a significant trade-off relationship with water yield and a significant synergistic relationship with soil conservation in the SHMB. In addition to the synergistic relationship between water yield and soil conservation (*p* < 0.05) in the SHMB under the RCP4.5 scenario in 2050, the correlation coefficients of soil conservation and water yield in 2010 and under the RCP8.5 scenario in 2050 were 0.018 and 0.017, respectively, which failed the significance test (*p* > 0.05). Significant synergies existed among water yield, NPP, and soil conservation in the CLPS, except for in 2010. A significant trade-off was present between water yield and NPP, and soil conservation also had significant synergies with water yield and NPP in the NEFB.

In terms of the temporal pattern, our results revealed that under both RCP scenarios, the synergy of water yield and NPP of the TSTB increased, whereas the relationship between soil conservation and water yield and between soil conservation and NPP of the TSTB were characterized by trade-offs. Each shelter presents different trade-offs and synergies under different RCP scenarios. Ecosystem services in the CLPS showed trade-offs and synergies consistent with the whole study area. Water yield and NPP, water yield and soil conservation, and NPP and soil conservation were characterized by synergies in the NEFB and NSPB under both RCP scenarios. A synergistic relationship was presented between water yield and NPP, whereas soil conservation and these two ecosystem services were characterized by trade-offs in the SHMB under the RCP4.5 scenario. Furthermore, water yield had a synergistic relationship with soil conservation, while there were trade-offs relationships between NPP and water yield and between soil conservation and NPP in the SHMB under the RCP8.5 scenario. The synergy of water yield and soil conservation of the TPES decreased, while NPP presented trade-offs with these two ecosystem services under the RCP4.5 scenario. The synergies of water yield, NPP and soil conservation in the TPES increased under the RCP8.5 scenario.

## 4. Discussion

### 4.1. Drivers of Changes in Ecosystem Services

**Figure 6:**
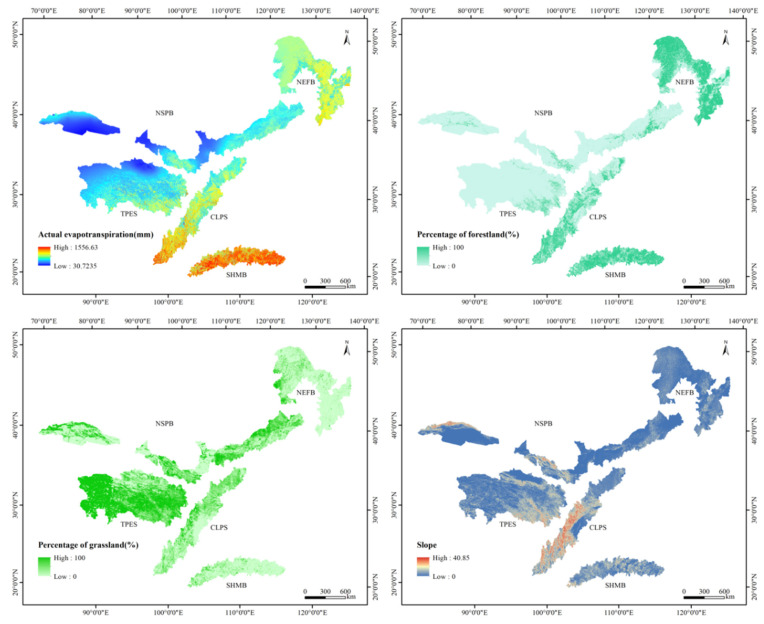
Spatial pattern of actual evapotranspiration, the percentage of forestland and grassland and slope in the TSTB.

Quantifying and mapping the spatial distribution of future ecosystem services are important steps to realize ecological management [63,64,65]. Under the combined action of natural factors and human activities, ecosystem services showed significant spatial differences. The main factors affecting ecosystem services are climate factors and land use factors [16,66]. In terms of spatial distribution, water yield is closely related to precipitation and actual evapotranspiration. Moreover, according to the water balance, water yield is determined by precipitation and actual evapotranspiration. Owing to the influence of water vapor from the southeast, the SHMB has abundant precipitation, and the spatial pattern shows a trend of decreasing from east to west. Although the region has relatively high actual evapotranspiration under the influence of multiple factors such as vegetation, temperature, and wind speed, the water yield of the region is relatively high owing to the replenishment of sufficient precipitation (Figure 6). However, the NEFB has less precipitation and relatively high actual evapotranspiration, so it has a lower water yield. The correlation analysis of water yield, precipitation, and actual evapotranspiration showed that precipitation is the main driving factor (r > 0.934; *p* < 0.01) for water yield (Table 4). This result was consistent with the results of previous studies [28,50,67]. However, the results were different from those of Wang and Dai [62]; our results indicated that actual evapotranspiration is positively correlated with water yield (r > 0.514; *p* < 0.01). In addition, the spatial distribution of water yield is similar to that of actual evapotranspiration. Although the findings emphasize the driving effect of precipitation, the contribution of other factors such as actual evapotranspiration to spatial differentiation of water yield cannot be denied. Similarly, Shirmohammadi found that the prediction result on the future water yield in the Urmia lake region was mainly sensitive to precipitation and evapotranspiration, especially precipitation [16]. Therefore, predicting the change of precipitation can be used to reveal the variation trend of water yield.

Under the influence of the average water yield and the distribution area, the water yield varied greatly among different land use types. Forestland and grassland are the main land use types in the TSTB, and the percentage of landscape is one of the land use factors affecting the water yield. Based on this, we analyzed the correlation between water yield and the percentage of forestland and grassland. The percentage of forestland and grassland were obtained through the Fragstats software (Figure 6). The results showed that the water yield was positively related to forestland (r > 0.376; *p* < 0.01), and negatively related to grassland (r < −0.273; *p* < 0.01), which indicated that the water yield was distributed in the area with a high proportion of grassland. This might have been related to the interception of precipitation in the forest canopy and the water holding capacity of the litter layer. Therefore, forestland is the main contributor to the water yield in the TSTB. In addition, the percentage of forestland is related to ecological restoration. Owing to the differences in evapotranspiration capacity, soil water content, litter water holding capacity and canopy interception of different land use types, there are significant differences in water yield among different land use types [68]. In general, the water yield is inversely related to the evapotranspiration of vegetation. Construction land and other land have little vegetation and evapotranspiration; therefore, the water yield capacity is high [69]. However, other land was located in the dry areas with low precipitation, which led to a low water yield. The evapotranspiration capacity of farmland varies greatly owing to the difference in crop types. The evapotranspiration capacity of water bodies was the strongest, which is clearly higher than that of vegetation. Therefore, the water yield of water bodies was the lowest, and a clear low-value area was formed in spatial distribution. A previous study found that the water yield of grassland is lower [70], but our results were different. The high water yield of forestland in the TSTB was mainly caused by its location near the area with high precipitation, and the water yield of forestland is greatly affected by precipitation. Therefore, the management policy of the TSTB needs to focus on ecological restoration.

The distribution of NPP was positively correlated with precipitation (r > 0.674; *p* < 0.01), temperature (r > 0.398; *p* < 0.01) and proportion of forestland (r > 0.803; *p* < 0.01), but negatively related to the proportion of grassland (r < −0.431; *p* < 0.01). This was consistent with the distribution trend of their high-value areas in the southeast and low-value areas in the northwest. Soil conservation had a positive correlation with precipitation (r > 0.220; *p* < 0.01), slope (r > 0.502; *p* < 0.01) and the proportion of forestland (r > 0.153; *p* < 0.01), but a negative correlation with grassland (r < −0.042; *p* < 0.01). The result showed that soil conservation was higher in places with greater precipitation. The results also highlight the impact of slope on soil conservation and the soil conservation was high in areas with a steep slope. Unlike the results of Wang and Dai [62], our results indicated that soil conservation was high in areas with a high proportion of forestland. The canopy has an interception effect on precipitation and vegetation roots can effectively intercept surface runoff and sediment, thereby reducing soil erosion and increasing soil conservation.

Global changes have significantly affected the supply of ecosystem services and seriously threatened the living environment of humans and the sustainable development of the social economy [71,72]. The land use change of the TSTB varies greatly. However, the proportion is very small, thereby indicating that the TSTB are affected by climate change. In terms of temporal changes, the water yield was positively correlated with precipitation (r > 0.862; *p* < 0.01) under both RCP scenarios from 2010 to 2050 (Table 5), which showed that precipitation was the main driving factor affecting water yield [67]. The increased precipitation led to the increased water yield, and under RCP8.5 scenario, more precipitation resulted in a higher water yield. However, the water yield was negatively correlated with actual evapotranspiration under the RCP4.5 scenario (r = −0.288; *p* < 0.01), but positively correlated with actual evapotranspiration under the RCP 8.5 scenario (r = 0.031; *p* < 0.01). The results indicated that the contribution of actual evapotranspiration to the change in water yield was different under different RCP scenarios. In the Taro Basin, water yield increased during the period 2010–2020 but decreased during the period 2030–2050 [15]. Our results also showed that the trend of water yield varied in different regions. Therefore, it is necessary to conduct extra studies in different regions to assess changes in water yield.

**Figure 7:**
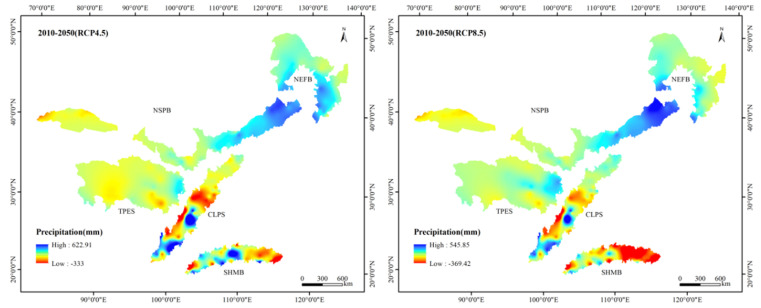
Spatial difference between 2010 and 2015 of precipitation in the TSTB.

The correlation analysis of temporal changes showed that temperature rise could increase NPP under both RCP scenarios (r > 0.191; *p* < 0.01). However, unlike the study conducted by Wang and Dai [62], our results showed that NPP had no significant relationship with precipitation under the RCP4.5 scenario, but a positive correlation with precipitation under the RCP8.5 scenario (r = 0.098; *p* < 0.01). Under both RCP scenarios, precipitation, temperature, and NPP all showed an upward trend, and NPP was slightly higher under RCP8.5 scenario. Feng revealed that NPP would increase under future climate change [30]. In addition to precipitation and temperature, NPP was also affected by ecological restoration policies. The impact of some policies on ecosystem services should not be ignored. Some studies showed that there were ecological restoration policies in the TSTB [44,45]. The implementation of an ecological restoration project significantly improved the vegetation coverage [44,45]. Moderate increases in temperature and precipitation contributed to vegetation coverage and productivity [44]. Zhu simulated the global terrestrial ecosystem NPP on the basis of each RCP scenario of CMIP5, and the increase in NPP was in direct proportion to the warming amplitude, especially in the southeastern United States, Central Africa, Southeast China and Western Amazon rainforest [73]. The research indicated that the total increase in terrestrial ecosystem NPP was mainly driven by the increase in atmospheric CO_2_ concentration, while other environmental factors had relatively weak influence [73]. Therefore, changes in NPP can be a good indicator of global climate change, and the assessment of NPP in the future is of great significance for predicting and evaluating the impact of the global carbon cycle on the development of human society in the future and formulating scientific climate policies. A significant positive correlation existed between soil conservation and precipitation (r > 0.314; *p* < 0.01). However, our results showed that under both RCP scenarios, precipitation increased while soil conservation declined. This implied that there were other factors contributing to the decrease in soil conservation, such as land use types and the spatial distribution of precipitation. The decrease in precipitation in the south resulted in the decrease in soil conservation, while the increase in precipitation in the north resulted in the increase in soil conservation (Figure 7). In addition, the decrease in precipitation in forestland led to the decrease in soil conservation and the increase in precipitation in grassland led to the increase in soil conservation. Although the total precipitation had an increasing trend, the spatial distribution showed different trends, thereby resulting in a possible downward trend in soil conservation. According to the RUSLE equation, a decrease in soil conservation also means a decrease in soil erosion, which indicates that soil erosion in the SHMB and CLPS will be alleviated to some extent in the future. In addition, the study of Ma on four basins in Central Asia showed that the soil conservation maintained a downward trend in the future under global warming scenarios [74], which is consistent with our study. The soil conservation in Central Asia is closely related to temperature and precipitation, while the soil conservation in the TSTB is closely related to slope and precipitation. Therefore, it is necessary to consider topographic factors when analyzing the spatial distribution of soil conservation.

In the Urmia lake region, land use exhibited considerable impact on the ecosystem services [16]. However, in the TSTB, land use changes are less pronounced and climate impacts may exceed these easily. Therefore, differences in the impact of land use change and climate change on ecosystem services depend on the environment. In general, the ecosystem services change in the barrier area is affected by climate change, and the ecological restoration also plays a certain role in the regional ecosystem services. Combined with climate change and ecological restoration, the provision of many ecosystem services is likely to change in the future, affecting the health and well-being of local populations [75]. Increased water yield may lead to flooding, reduce the accessibility of green space, and reduce the recreational health benefits [76]. Ecological restoration can increase vegetation cover, reduce air pollution, improve air quality, and improve respiratory related infections/diseases, with a beneficial impact on the aesthetics of the local environment [77].

### 4.2. Spatial Relationship among Ecosystem Services

Our results demonstrated the synergies among water yield, NPP, and soil conservation in terms of spatial distribution, which was consistent with the results of Yin [46]. However, there was a significant trade-off relationship (r < −0.196, *p* < 0.01) between water yield and NPP in the SHMB and NEFB owing to precipitation and vegetation drivers. Similar studies have shown that the increase in NPP reduces water yield by expanding vegetation evapotranspiration [78]. However, except for CLPS in 2015, water yield and NPP showed a synergistic relationship in other shelters. The different relationship between water yield and NPP was related to geographical differences. In terms of temporal variation, with the combination of climate change and ecological restoration, there was a synergistic relationship between water yield and NPP, and the relationships between water yield and soil conservation and between NPP and soil conservation were characterized by trade-offs. Wang showed that there was a trade-off between water yield and NPP in terms of temporal variation owing to the implementation of GFGP [33]. However, from the perspective of land use, it is difficult to observe the significant GFGP in the TSTB, which caused different results. Our results indicated the complex relationships among ecosystem services and highlighted the importance of these relationships in ecosystem management. Therefore, ecological management should consider future climate change.

Climate change and policy implementation often lead to tradeoffs in ecosystem services, such as studies in Western North Carolina, Mediterranean forests and Guayas River Basin [79,80,81]. Coupled models of climate change, land use change, and ecosystem services can help to understand the impacts and trade-offs of climate change and policy. By revealing ecosystem service tradeoffs, decision makers can reduce or eliminate these ecosystem service trade-offs by adjusting land use patterns. Our results also suggest that there are scale effects on ecosystem service trade-offs. By quantifying the ecosystem service trade-offs and analyzing the main factors affecting ecosystem services in each shelter area, effective ecological management measures can be developed for different regions.

### 4.3. Different Management Strategies

Grassland is the main land use type in NSPB and TPES, which is significantly affected by temperature and precipitation [44,82]. Therefore, the ecosystem management in TPES and NSPB should consider the fragile environment and focus on the ecological protection, especially the protection of grassland. The Loess Plateau ecological shelter is located in the arid and semi-arid region and the growth of plantation vegetation requires a large amount of water, which will aggravate the water shortage of CLPS [28,44]. In addition, the increase in CLPS water yield from 2010 to 2050 was not significant, and water stress still existed. Thus, the ecological construction in CLPS need to pay more attention to the rational use of water resources to ensure the water use of the barrier area. There is always a trade-off between water yield and NPP in NEFB and SHMB. It is necessary to weaken or eliminate the trade-off in making ecological restoration policy based on spatial trade-off. SHMB enjoys favorable climatic conditions and forest ecosystem services are superior to other ecosystems [44,83]. SHMB has a large area of farmland, which is an important place for production activities and faces the pressure of economic development. This means the contradiction between ecological protection and economic development will coexist for a long time [44]. Therefore, it is necessary for the ecological construction of SHMB to comprehensively consider ecological protection and economic development and develop sustainable ecological management strategies on this basis. Strictly, expansion of cities should be controlled, and permanent farmland should be established to ensure food security. The TSTB are important components of the national ecological security strategy in the construction of ecological civilization and lay a solid foundation for national ecological security. The purpose of constructing TSTB in the ecological security strategy pattern is to improve ecosystem services and guarantee China’s ecological security. Our results can provide a scientific basis for sustainable ecosystem management in shelter areas in response to future climate change.

### 4.4. Limitations and Future Research

In this study, ecosystem services and their relationships were evaluated in the TSTB as a whole. However, the characteristics of each shelter were different, and the main ecosystem services were also different. The ecosystem services we selected do not fully represent the ecosystem services of the shelters. Future research must conduct a comprehensive and detailed assessment of the shelters and in-depth analysis of the trade-offs and synergies in order to better optimize the management and formulate ecological protection policies according to local conditions. Only one CMIP5 climate model (BNU-ESM) and two RCP scenarios were used in this study. However, there are many climate models, and the simulation accuracy of different models is different, so it is difficult to fully reflect the future climate change. More climate models and RCP scenarios need to be considered in the future. In the context of global changes, future development is full of uncertainties, so it is necessary to explore the trade-offs and synergies under different development scenarios in the future in order to seek a win–win situation of economic development and environmental protection and provide a reference for the government’s ecological planning and construction.

The ecological restoration scenario was designed using the FLUS model, but the uncertainty related to the spatial model cannot be ignored. Land use simulations are based on historical rates of change, ignoring potential changes in human activity and socio-economic development, and so there may be great uncertainty about future land use. Therefore, future research should pay more attention to integrated land use history and demographic trends. In addition, time-dependent uncertainty analysis can be used to further explore and quantify the uncertainties of input parameters and model structure. Sensitivity analysis and other methods can identify the source of uncertainty, which is helpful to optimize the output of the model. Land use and climate change can influence each other, but this study did not consider the impact of future climate change when simulating land use change. To better simulate future scenarios, future research should focus on the response of land use change to climate change. In addition, this study only considers the ecological restoration scenario. More ecological environmental and socio-economic factors, such as ecological restoration implementation intensity and urbanization, should also be considered in a scenario setting to make the simulation scenario more comprehensive and the results more instructive for regional policy making.

In this study, due to the lack of field survey data, the spatial distribution data from other studies were used for model calibration. This may result in some uncertainty or bias in ecosystem service assessment results in 2015, but no significant errors at the overall level. However, the results of the 2050 ecosystem services assessment remain highly uncertain. In addition, the model itself is uncertain. The simulation accuracy of the model is related to many factors, which cannot fully participate in the modeling process, so it is inevitable that there will be some cases that are not considered. For example, the water yield module in InVEST model does not consider the impact of topography and groundwater on water production, which will lead to errors in the results. Moreover, we used the 1 km resolution land use data provided by the Resources and Environment Data Center of the Chinese Academy of Sciences. Although the age and resolution of the data are not strongly relevant to the objectives of this study, the use of up-to-date and high-resolution data will further reduce the uncertainty of the results.

## 5. Conclusions

In this study, we quantified future ecosystem services under two climate scenarios and identified trade-offs and synergies among ecosystem services through statistical analysis. Overall, the changes in ecosystem services under both RCP scenarios showed a consistent trend, but changes in ecosystem services under the RCP8.5 scenario were larger than those under the RCP4.5 scenario. Water yield, NPP, and soil conservation all showed a spatial pattern of decreasing from southeast to northwest, while in terms of the temporal pattern, water yield and NPP increased, but soil conservation decreased. The spatial and temporal variations in water yield were mainly controlled by rainfall, while the variations in NPP were influenced by temperature and the implementation of ecological restoration. The driving factors of soil conservation changes were rainfall and slope. Overall, synergies existed among water yield, NPP, and soil conservation in terms of spatial trends. There was strong spatial heterogeneity in trade-offs and synergies. Under different RCP scenarios, the trade-offs and synergies were slightly different. In terms of temporal pattern, with the combination of climate change and ecological restoration, there was a synergistic relationship between water yield and NPP, and the relationships between water yield and soil conservation and between NPP and soil conservation were characterized by trade-offs. Ecosystem service trade-offs and synergies differ at different scales. The formulation of ecological protection policy needs to consider local characteristics and climate change. Under future climate change, an increase in NPP will benefit the health and well-being of the local population, while an increase in water yield may reduce the recreational benefits of health. In addition, ecological remediation plays an important role in reducing air pollution, tourism and recreational activities, helping to improve air quality and respiratory related infections/diseases. This study can provide land managers and decision makers with feasible paths for sustainable ecosystem management under climate and land use change in the future. This study can also provide a reference for assessing the future development trajectories of other regions to better achieve the balance between socio-economic development and environmental sustainability.

## Figures and Tables

**Table 1:** The composition of land use types in the TSTB and each shelter in 2010 and 2050 (km^2^).

		Farmland	Forestland	Grassland	Construction Land	Water Body	Other Land
TSTB	2010	407,536	893,421	1,233,191	66,960	24,334	534,669
	2050	409,596	900,283	1,217,817	68,904	29,597	533,914
CLPS	2010	127,733	196,259	150,871	3971	5489	3782
	2050	128,198	199,017	147,149	3928	6059	3754
SHMB	2010	50,268	201,831	36,132	2768	2629	199
	2050	50,292	202,297	35,385	3128	2526	199
TPES	2010	2946	42,011	650,236	36,356	553	194,255
	2050	2946	42,084	647,165	38,315	2083	193,764
NEFB	2010	109,711	391,015	80,531	6446	5661	20,389
	2050	109,196	395,380	77,326	5969	6370	19,512
NSPB	2010	116,878	62,305	315,421	17,419	10,002	316,044
	2050	118,964	61,505	310,792	17,564	12,559	316,685

TSTB: two ecological shelters and three belts. CLPS: Chuan-Dian and Loess Plateau ecological shelter. SHMB: southern hill and mountain belt. TPES: Tibet Plateau ecological shelter. NEFB: northeast forest belt. NSPB: northern sand prevention belt.

**Table 2:** The average water yield, net primary productivity (NPP), and soil conservation in the TSTB and each shelter in 2010 and 2050.

		TSTB	CLPS	SHMB	TPES	NEFB	NSPB
water yield (mm)	2010	208.04	406.47	847.85	115.17	124.09	31.09
	2050 RCP4.5	235.93	414.95	906.88	102.18	198.29	70.69
	2050 RCP8.5	232.92	423.9	802.39	119.4	182.91	83.11
NPP (cg/m^2^)	2010	263.17	410.87	522.5	120.73	431.27	119.25
	2050 RCP4.5	303.09	509.85	603.93	146.77	455.45	137.10
	2050 RCP8.5	303.15	513.05	607.63	147.41	450.60	137.05
soil conservation (t/km^2^)	2010	3089.00	11,119.87	6008.5	1771.59	703.68	571.52
	2050 RCP4.5	3078.12	10,885.72	6001.52	1753.94	810.38	610.05
	2050 RCP8.5	3070.17	10,863.15	5574.79	1875.88	785.25	626.67

TSTB: two ecological shelters and three belts. CLPS: Chuan-Dian and Loess Plateau ecological shelter. SHMB: southern hill and mountain belt. TPES: Tibet Plateau ecological shelter. NEFB: northeast forest belt. NSPB: northern sand prevention belt.

**Table 3:** The correlation coefficients among water yield, NPP, and soil conservation in 2010 and under the two RCP scenarios in 2050.

		2015	2050 RCP4.5	2050 RCP8.5
TSTB	water yield and NPP	0.484 **	0.531 **	0.536 **
	water yield and soil conservation	0.199 **	0.188 **	0.186 **
	NPP and soil conservation	0.208 **	0.227 **	0.227 **
CLPS	water yield and NPP	−0.014	0.093 **	0.052 **
	water yield and soil conservation	0.149 **	0.15 **	0.125 **
	NPP and soil conservation	0.194 **	0.188 **	0.186 **
SHMB	water yield and NPP	−0.302 **	−0.293 **	−0.263 **
	water yield and soil conservation	0.018	0.026 *	0.017
	NPP and soil conservation	0.103 **	0.073 **	0.079 **
TPES	water yield and NPP	0.584 **	0.593 **	0.586 **
	water yield and soil conservation	0.227 **	0.233 **	0.215 **
	NPP and soil conservation	0.344 **	0.354 **	0.353 **
NEFB	water yield and NPP	−0.278 **	−0.196 **	−0.216 **
	water yield and soil conservation	0.073 **	0.098 **	0.102 **
	NPP and soil conservation	0.145 **	0.178 **	0.178 **
NSPB	water yield and NPP	0.349 **	0.461 **	0.482 **
	water yield and soil conservation	0.077 **	0.029 **	0.027 **
	NPP and soil conservation	0.120 **	0.150 **	0.167 **

Note: ** *p* < 0.01, * *p* < 0.05.

**Table 4:** Correlation between ecosystem services and impact factors for spatial changes.

	water yield		
	2010	2050 RCP4.5	2050 RCP8.5
precipitation	0.941 **	0.947 **	0.934 **
actual evapotranspiration	0.575 **	0.542 **	0.514 **
percentage of forest	0.376 **	0.412 **	0.386 **
percentage of grass	−0.273 **	−0.309 **	−0.278 **
	NPP		
	2010	2050 RCP4.5	2050 RCP8.5
precipitation	0.674 **	0.700 **	0.716 **
temperature	0.398 **	0.455 **	0.469 **
percentage of forest	0.834 **	0.805 **	0.803 **
percentage of grass	−0.449 **	−0.432 **	−0.431 **
	soil conservation		
	2010	2050 RCP4.5	2050 RCP8.5
precipitation	0.220 **	0.248 **	0.218 **
slope	0.506 **	0.502 **	0.506 **
percentage of forest	0.153 **	0.159 **	0.154 **
percentage of grass	−0.037 **	−0.042 **	−0.037 **

Note: ** *p* < 0.01.

**Table 5:** Correlation between ecosystem services and impact factors for temporal changes.

	water yield	
	2010–2050 RCP4.5	2010–2050 RCP8.5
precipitation	0.862 **	0.883 **
actual evapotranspiration	−0.288 **	0.031 **
	NPP	
	2010–2050 RCP4.5	2010–2050 RCP8.5
precipitation	−0.050	0.098 **
temperature	0.280 **	0.191 **
	soil conservation	
	2010–2050 RCP4.5	2010–2050 RCP8.5
precipitation	0.338 **	0.314 **

Note: ** *p* < 0.01.
